# Improving Patient Safety Culture During the COVID-19 Pandemic in Taiwan

**DOI:** 10.3389/fpubh.2022.889870

**Published:** 2022-07-12

**Authors:** Shu Jung Wang, Yun Chen Chang, Wen Yu Hu, Yang Hsin Shih, Ching Hsu Yang

**Affiliations:** ^1^School of Nursing, College of Medicine, National Taiwan University, Taipei, Taiwan; ^2^School of Nursing and Graduate Institute of Nursing, China Medical University, Taichung, Taiwan; ^3^Department of Nursing, National Taiwan University Hospital, Taipei, Taiwan; ^4^Superintendent Office, Central Clinic & Hospital, Taipei, Taiwan; ^5^Department of Emergency Medicine, Hsinchu Mackay Memorial Hospital, Hsinchu, Taiwan

**Keywords:** patient safety culture, COVID-19, emotional exhaustion, work life balance, patient safety

## Abstract

**Background and Aim:**

Patient safety culture attitude is strongly linked to patient safety outcomes. Since the onset of the COVID-19 pandemic in early 2020, pandemic prevention has become the priority of hospital staff. However, few studies have explored the changes in patient safety culture among hospital staff that have occurred during the pandemic. The present study compared the safety attitudes, emotional exhaustion (EE), and work–life balance (WLB) of hospital staff in the early (2020) and late (2021) stages of the COVID-19 pandemic and explored the effects of EE and WLB on patient safety attitudes in Taiwan.

**Materials and Methods:**

In this cross-sectional study, the Joint Commission of Taiwan Patient Safety Culture Survey, including the six-dimension Safety Attitudes Questionnaire (SAQ) and EE and WLB scales, were used for data collection.

**Results:**

This study included a total of 706 hospital employees from a district hospital in Taipei City. The respondents' scores in each SAQ sub-dimension (except for stress recognition) increased non-significantly from 2020 to 2021, whereas their EE and WLB scores improved significantly (*P* < 0.05 and *P* < 0.01, respectively). The results of hierarchical regression analysis indicated that although a respondent's WLB score could predict their scores in each SAQ sub-dimension (except for stress recognition), EE was the most important factor affecting the respondents' attitudes toward patient safety culture during the later stage of the COVID-19 pandemic.

**Conclusion:**

In the post-pandemic, employees' attitudes toward safety climate, job satisfaction, and perception of Management changed from negative to positive. Additionally, both EE and WLB are key factors influencing patient safety culture. The present study can be used as a reference for hospital managers to formulate crisis response strategies.

## Introduction

The challenges to patient safety due to the COVID-19 outbreak, such as an imbalance in the supply and demand of protective equipment, rapid changes in policies, lack of evidence-based treatment guidelines for COVID-19, and inadequate supervision of procedures due to lack of personnel, make it easy to make mistakes (1). In response to this crisis, workers are on guard to improve safety behaviors (2). However, risk perception can increase anxiety and negatively affect safety performance. Research has Indicated that a team safety climate can alleviate this negative psychological impact (3). Safety climate is often used interchangeably with safety culture, with the difference being that the former refers to the stable characteristics of the organization. At the same time, the latter is the state of the environment at a given time (4). The favorable safety climate during SARS 2003 was also an organizational factor in protecting hospital staff from infectious diseases (5).

Adverse events during hospitalization affect one in 10 hospitalized patients (6). These events are associated with surgery (27%), medication errors (18.3%), and nosocomial infections (12.2%). Approximately 53.2% of these events are preventable (7). Ensuring patient safety and optimizing the provision of medical care by health-care professionals are essential to promoting high-quality health care. Patient safety and risk management training enhances staff adherence to patient safety, thus building a safety culture (8). Making efforts to foster a culture of safety is key to improving patient safety and the quality of care in nursing settings (9). The significance of the culture of safety as the sustainable approach to fostering safety has been emphasized by most health organizations such as the World Health Organization and Joint Commission International (JCI), which are international authorization associations (10). A system of patient safety culture can be constructed by drawing on the shared values, beliefs, norms, and patient safety procedures among the members of a health-care organization, unit, or team (11, 12). Safety culture can established from the effective interaction of three components: (a) environmental structures and processes within an organization, (b) worker attitudes and perceptions, and (c) individual behaviors related to safety (13).

Effective patient safety culture can decrease mortality to 44,000 and can reduce economic loss to US$2.9 billion (14, 15). In addition, it can facilitate the implementation of improved safety measures, promote effective communication, and encourage individuals to learn from their mistakes (16). Accordingly, it can reduce fatigue and psychological and work-related stress among employees and can promote their health and job performance. Overall, studies have demonstrated that positive patient safety culture contributes positively to patient satisfaction, family satisfaction, and the wellbeing of hospital staff and can even decrease hospital admissions (17, 18).

Organizational safety culture signifies “the outcome of the values, attitudes, competencies, and behavioral patterns of individuals and groups that ascertain commitment, style and efficiency in the management of an organization's health and safety. The features of a positive safety culture are communications based on mutual trust, a shared understanding of the importance of safety, and confidence in the effectiveness of precautionary measure” (19).

Due to the increasing awareness of the importance of hospital-wide patient safety culture, tools have been developed to assess the safety attitudes of hospital staff. Among the numerous cognitive tools to evaluate employee attitudes toward safety in health-care facilities, the most frequently used is the Safety Attitudes Questionnaire (SAQ) (20). The SAQ has undergone numerous revisions to improve its precision and ability to meet the needs of different units within a health-care organization (21).

The Joint Commission of Taiwan (JCT; https://www.jct.org.tw/cp-21-1155-4a85d-1.html), founded in 1999, is a professional assessment institute accredited by the International Society for Quality in Health Care (ISQua). In Taiwan, the SAQ is used to conduct an annual national survey to monitor long-term trends in patient safety culture (22, 23). The questionnaire accounts for six aspects of patient safety culture (namely teamwork ethos, safety ethos, job satisfaction, stress recognition, perception of management, and work conditions) and exhibits high internal consistency (Cronbach's α = 0.78) (24). The JCT incorporated scales evaluating work–life balance (WLB) and emotional exhaustion (EE) into its annual patient safety culture survey in 2014 to detect burnout and work–life imbalance among hospital staff to eliminate their negative effects on patient safety culture.

Health-care workers, including nurses and those working in non-emergency wards of hospitals, are under great pressure as they are more vulnerable to COVID-19 (25). Throughout the COVID-19 pandemic, health-care professionals have experienced problems in terms of limited hospital resources, the threat of exposure to SARS-CoV-2 as an additional occupational hazard, increased workloads, fear of transmitting COVID-19 to family members, and disrupted sleep patterns, leading some to become agitated or even commit suicide (26). Although the death toll of COVID-19 in Taiwan (a total of 850 deaths as of December 29, 2021) has remained low relative to that in other countries. As a frontline medical worker, employment must deal with patient emotions and do related coordination under the epidemic's limited social contact policy, including restricting elective surgery or hospitalization and patient visits, which are likely ethical issues affecting patient autonomy (27). Meanwhile, because they must have close interaction with infected patients, may result in psychological and emotional trauma, acute stress disorder, and post-traumatic stress disorder (26). In addition, significant correlations have been identified among the work environment, EE, depersonalization (an alienated or apathetic attitude toward work), personal achievement, and organizational patient safety culture (28).

Work–life balance is based on the allocation of available personal resources. WLB is achieved when an individual's personal resources are sufficient for their professional and familial roles, thereby enabling them to effectively participate in each area (29). During the COVID-19 pandemic, a long-term work–life imbalance has resulted in high rates of burnout among medical staff. An individual's WLB affects not only the quality of professional life and family life but also affects the overall quality of life (30). The relationships between WLB, resilience, and patient safety culture have not been thoroughly explored.

Senior leadership accountability (31), teamwork within a hospital, and organizational learning strongly affect organizational safety culture (32). The impact of COVID-19 on patient safety culture has been previously studied (22, 33); as of 2022, the COVID-19 pandemic has extended into its third year, and how the patient safety culture has adapted from various problems over time, such as personal protective equipment shortage, insufficient resources, increased costs and reduced revenue, and often-changing central policies in the early days of the outbreak (34), especially in district hospitals with relatively. However, no study has explored patient safety culture in district hospitals. For addressing this research gap, the study evaluated the differences in patient safety culture between the early (2020) and late (2021) stages of the COVID-19 pandemic in a district hospital in Taiwan and explored the effects of WLB and EE on SAQ subdimension scores.

## Materials and Methods

### Study Design

This study employed a cross-sectional design. The original file (Microsoft Excel file) containing the results of the 2020 and 2021 patient safety culture surveys of a hospital in Taipei (2020, *N* = 363; 2021, *N* = 343) was used as the data source. The data were collected from a district hospital with fewer than 200 beds. Every August, the hospital administration conducts routine patient safety culture surveys for employees who have worked at the hospital for more than 3 months.

### Data Collection

The test schedule was announced before the survey. During the test period, the supervisor was requested through the hospital Line group or at a hospital executive meeting) to encourage eligible employees to fill out the questionnaire. Employees could fill out the questionnaire online by clicking a link sent to them over email. Employees without email addresses were provided with a separate account and password on paper to access the online questionnaire. Some staff filled the questionnaire in paper form, which was sent to the undertaker in an official document and keyed into the file. All the questionnaires were anonymous; no identifiable personal information (such as account numbers or personal emails) was included in the data imported from the questionnaire. In this way, the survey answers go directly to an external system (JCT Patient Safety Culture Platform), eliminating the stress on supervisors when filling out the questionnaires. Therefore, colleagues are better able to respond to the survey based on their accurate perceptions and awareness.

### Instruments

#### Demographic

We collected the following baseline demographic and professional information for each respondent: age, gender, educational level, tenure, profession, division, managerial status, number of incidents submitted in the past 12 months, and whether they have contact with patients at work.

#### SAQ

The SAQ (21) was translated into Chinese by Dr. Lee Wai-keung in Taiwan (with the permission of Dr. Sexton JB of the University of Texas), and it has been incorporated into the national surveys which was conducted annually by JCI. The questionnaire contains 30 items across six sub-dimensions: teamwork climate, safety climate, job satisfaction, stress recognition, perception of management, and working conditions. Each item on the questionnaire is rated on a 5-point Likert-scale (1 = strongly disagree, 2 = slightly disagree, 3 = neutral, 4 = slightly agree, and 5 = agree). Not applicable responses are scored as 0 points. A respondent's SAQ sub-dimension score is calculated as (dimension mean score −1) × 25 and is regarded as a positive attitude if it is ≥75. The SAQ is widely used in many countries, with Cronbach's α values ranging from 0.85 (35) to 0.88 (36), indicating its high internal consistency and reliability. In the present study, the Cronbach's α values of the sub-dimensions ranged from 0.83 to 0.91, indicating the scale's high internal consistency and reliability (Table 1).

**Table 1:** Internal consistency reliability of the SAQ.

**Dimension**	**Sub-dimension**	**Definition (21)**	**Item**	**Cronbach's** ***α***	
				**2020**	**2021**
SAQ	Teamwork climate	Perceived quality of collaboration between personnel	6	0.85	0.85
	Safety climate	Perceptions of a strong and proactive organizational commitment to safety	7	0.88	0.90
	Job satisfaction	Positivity about the work experience	5	0.93	0.95
	Stress recognition	Acknowledgment of how performance is influenced by stressors	4	0.88	0.86
	Perception of management	Approval of managerial action	4	0.83	0.91
	Working condition	Perceived quality of the work environment and logistical support (staffing, equipment etc.)	4	0.87	0.84

#### EE Questionnaire

In addition to the SAQ, this study used the EE component of the Maslach Burnout Inventory developed by Maslach et al. in 1976 (37). The scoring of the EE scale is the same as that of the SAQ. The Cronbach's alpha values for the 2020 and 2021 questionnaires were 0.90 and 0.91, respectively.

#### WLB Questionnaire

The 7-item College Activities and Behavior Questionnaire by Sexton et al. (21)was adapted for use in health-care professionals as the WLB questionnaire in this study. Each item on the WLB questionnaire is rated on a 4-point Likert-scale almost never, less than 1 day per week), 4 points; sometimes (1–2 days per week), 3 points; most of the time (3–4days per week), 2 points; and always (5–7 days per week), 1 point. Not applicable responses are scored as 0 points. A respondent's total WLB score is calculated as (dimension mean score −1) × 33.3 and is regarded as positive if it is ≥63.3. The Cronbach's alpha values for the 2020 and 2021 questionnaires were 0.83 and 0.82, respectively.

### Data Analysis

Statistical analyses were used the SPSS 25.0 software package, and the distribution of basic employee data was obtained from descriptive statistics (means, standard deviations, frequencies, and percentages). One-way analysis of variance (ANOVA) and independent *t*-tests were used for bivariable analysis of demographic and professional variables and SAQ score, EE, and WLB. Spearman's correlation co-efficient was used to identify the correlations among the SAQ subdimension, EE, and WLB scores. Hierarchical regression analysis was performed to predict the power of demographic and professional variables and EE and WLB scores for patient safety culture (SAQ sub-dimensions).

### Compliance With Ethical Standards

Although no personal information was included in the study data, the data were still treated as confidential and will not be disclosed. All the identifiable information in our data has been replaced with codes and all the electronic files and documents related to the study are protected and encrypted. Only the research team members can access the research-related materials, and all the research-related materials will be destroyed after the research results are published.

## Results

### Demographics and Characteristics

A total of 343 valid 2021 questionnaires were collected. Most (80.2%) of the respondents were women, most of whom were nurses. Nearly 70% of the respondents were over 40 years old, and 18.7% were managers. Most of the respondents had a college degree or above (87.8%), and nearly 50% (45.5%) had worked in the hospital for more than 10 years. A total of 79.3% of the respondents reported that they have contact with patients during their daily work, and 19% described that they had reported an incident within the preceding 12 months. The respondents' basic information in the 2021 questionnaire was the same as their information in the 2020 questionnaire, with no significant differences revealed by the chi-squared test (Table 2).

**Table 2:** Demographic and clinical characteristics.

**Characteristics**	**2020 year (*****n*** **=** **363)**		**2021 year (*****n*** **=** **343)**		**χ^2^**	***p-value***
	***n***	***%***	***n***	***%***		
**Age group**						
20–40 years	126	34.7	115	33.5	0.450	0.799
40–60 years	165	45.5	153	44.6		
≥60 years	72	19.8	75	21.9		
**Gender**						
Male	84	23.1	68	19.8	1.147	0.314^a^
Female	279	76.9	275	80.2		
**Educational level**						
High school or less	54	14.9	42	12.2	1.471	0.479
Diploma or Bachelor	272	74.9	270	78.7		
Master or doctor degree	37	10.2	31	9.0		
**Tenure**						
3 months-1 year	41	11.3	29	8.5	3.311	0.346
1–4 years	94	25.9	104	30.3		
5–10 years	66	18.2	54	15.7		
>10 years	162	44.6	156	45.5		
**Profession**						
Physician	50	13.8	33	9.6	3.190	0.363
Nurse	141	38.8	135	39.4		
Technician	55	15.2	53	15.5		
Administrative	117	32.2	122	35.6		
**Division**						
High risk department	137	37.7	119	34.7	0.837	0.658
OPD/Inspection units	160	44.4	162	47.2		
Administration units and others	66	18.2	62	18.1		
**Managerial position**						
Yes	62	17.1	64	18.7	0.300	0.623^a^
No	301	82.9	279	81.3		
**Incident reports**						
None	283	78.0	278	81.0	1.031	0.351^a^
At least one	80	22.0	65	19.0		
**Patient contact**						
No	62	17.1	71	20.7	1.511	0.248^a^
Yes	301	82.9	272	79.3		

^a^* Fisher's exact test*.

### Comparison of SAQ, EE, and WLB Scores in 2020 and 2021

As shown in Table 3, the average EE and WLB scores in 2021 were significantly higher than those in 2020 (*P* < 0.05 and *P* < 0.001, respectively). Among the SAQ, EE, and WLB scores, only the EE and WLB scores changed significantly from 2020 to 2021. The average stress recognition score in 2021 was slightly lower than that in 2020, but this change was not statistically significant (*P* > 0.05). Regarding the mean score, only the teamwork climate subdimension score was positive (≥75 points) in 2020, and in 2021, the safety climate, job satisfaction, and perception of management subdimension scores were all positive, except for the average teamwork climate subdimension score.

**Table 3:** SAQ, EE, and WLB (2020 vs. 2021).

**Dimension**	**2020**		**2021**		***t***	***p-value***
	***N***	**Mean (SD)**	***N***	**Mean (SD)**		
**SAQ total score**	363	71.41 (16.55)	343	72.44 (15.82)	0.846	0.398
Teamwork climate	344	76.18 (18.29)	325	78.50 (16.51)	1.719	0.086
Safety climate	356	74.38 (18.01)	334	76.37 (17.25)	1.483	0.138
Job satisfaction	362	74.28 (20.97)	343	76.00 (20.79)	1.095	0.274
Stress recognition	361	63.70 (24.44)	341	62.05 (22.37)	−0.933	0.351
Perception of management	361	74.64 (19.35)	342	75.43 (19.73)	0.532	0.595
Working condition	354	71.23 (20.60)	331	72.24 (19.12)	0.663	0.507
**Emotional exhaustion**	361	37.87 (19.48)	337	34.11 (20.47)	−2.488	0.013*
**Work-life balance**	337	54.80 (13.34)	343	57.56 (12.47)	2.846	0.005**

*P < 0.05, **P < 0.01.

### Changes in SAQ, EE, and WLB Scores Across Demographic Variables

To understand the factors affecting the respondents' SAQ, EE, and WLB scores in 2021, a bivariate analysis including demographic and professional variables, patient safety culture attitudes, EE, and WLB was conducted (see Table 4). The mean SAQ score differed across age groups (*P* = 0.001), and the mean total SAQ score of the ≥60 years age group was significantly higher than those of the other age groups.

**Table 4:** Bivariable analysis of demographic and SAQ, EE, WLB.

**Dimension**	***n***	**SAQ total**	**EE**	**WLB**
**Variable**		**Mean (SD)**	**Mean (SD)**	**Mean (SD)**
**Age group**				
^1^ 20–40 years	115	69.94 (15.46)	40.63 (18.50)	57.55 (11.04)
^2^ 40–60 years	153	71.93 (17.05)	32.74 (19.69)	57.24 (12.79)
^3^ ≥60 years	75	77.32 (12.56)	26.98 (22.22)	58.24 (13.94)
*P-*value		0.001**	<0.001***	0.851
*post-hoc*		3>1, 2	1>2, 3	
**Gender**				
Male	68	75.15 (13.72)	28.32 (19.44)	59.77 (11.70)
Female	275	71.77 (16.25)	35.52 (20.50)	57.01 (12.61)
*P-*value		0.115	0.010*	0.103
**Educational level**				
High school or less	42	72.48 (16.90)	32.40 (21.80)	60.29 (11.84)
Diploma or Bachelor	270	72.36 (15.69)	35.11 (20.58)	56.92 (12.54)
Master or doctor degree	31	73.07 (16.02)	27.69 (16.77)	59.45 (12.39)
*P-*value		0.973	0.138	0.179
**Tenure**				
^1^ 3 months-1 year	29	76.42 (17.48)	27.50 (21.97)	62.81 (11.51)
^2^ 1–4 years	104	72.17 (16.67)	31.78 (20.70)	59.65 (12.44)
^3^ 5–10 years	54	72.04 (13.43)	34.76 (19.48)	56.22 (13.51)
^4^ >10 years	156	72.03 (15.73)	36.66 (20.12)	55.65 (11.90)
*P-*value		0.573	0.082	0.006**
*post-hoc*				1>4
**Profession**				
^1^ Physician	33	75.26 (12.35)	27.00 (20.51)	56.28 (13.03)
^2^ Nurse	135	71.55 (15.32)	41.00 (18.24)	53.54 (12.57)
^3^ Technician	53	76.16 (14.38)	28.67 (17.98)	60.78 (9.67)
^4^ Administrative	122	71.05 (17.54)	30.66 (21.83)	60.95 (12.04)
*P-*value		0.108	<0.001***	<0.001***
*post-hoc*			2>1, 3, 4	3, 4>2
**Division**				
^1^ High risk department	119	69.30 (14.26)	41.14 (18.07)	51.89 (12.05)
^2^ OPD/inspection units	162	76.36 (14.54)	30.57 (19.62)	59.74 (12.28)
^3^ Administration units	62	68.24 (19.34)	29.70 (23.52)	62.73 (9.52)
*P-*value		<0.001***	<0.001***	<0.001***
*post-hoc*		2>1, 3	1>2, 3	2, 3>1
**Managerial position**				
Yes	64	73.34 (13.72)	37.19 (17.90)	51.95 (13.44)
No	279	72.24 (16.28)	33.41 (20.98)	58.85 (11.90)
*P-*value		0.576	0.187	<0.001***
**Incident reports**				
None	278	72.46 (16.07)	32.48 (19.67)	58.98 (12.14)
At least one	65	72.37 (14.83)	41.08 (22.44)	51.48 (12.11)
*P-*value		0.965	0.002**	<0.001***
**Patient contact**				
Yes	272	73.03 (15.14)	35.26 (20.34)	56.01 (12.56)
No	71	70.18 (18.15)	29.74 (20.53)	63.48 (10.21)
*P-*value		0.225	0.044*	<0.001***

*EE, emotional exhaustion; WLB, work-life balance*.

* P < 0.05, ** P < 0.01, ^***^ P < 0.001.

Regarding division, the employees who worked in outpatient/inspection units had higher SAQ scores (*P* <0.001) than did those who worked in high-risk units and administrative departments. Gender, educational level, tenure, profession, managerial status, number of incident reports, and whether they have contact with patients at work did not affect the overall SAQ score.

Regarding EE, the employees over 60 years old (*P* < 0.001) had the lowest mean EE score, and those 20–40 years old had the highest mean EE score. The men experienced less EE than did the women (*P* = 0.010). Regarding profession, the mean EE score of the nurses was significantly higher than those of the respondents in other professions. The physicians had the lowest mean EE score, but their mean EE score was not significantly different from those of the other medical technicians and administrative staff. As we had expected, the respondents who worked in high-risk units had the highest mean EE score, as we had expected. The average EE scores of the respondents employed in outpatient/inspection units and administrative units were not significantly different (*P* = 0.958).

The respondents who had reported at least one incident within the preceding 12 months had a higher mean EE score than did those who had not reported any incident (*P* = 0.002). The respondents who have no contact with patients in their daily work also had a lower mean EE score than did those who have contact with patients in their daily work (*P* = 0.044). Gender, educational level, tenure, and managerial status had no effect on the EE score.

The average WLB scores of most junior employees (those who had been employed for 3 months to 1 year) were significantly higher than those of most senior employees (those who had been employed for >10 years; *P* = 0.006). The mean WLB score of the nurses was lower than those of the other medical technicians (*P* = 0.004) and of the non-medical staff (*P* < 0.001), but the score of the nurses was comparable to that of the physicians (*P* = 0.713). The respondents who worked in outpatient/inspection units and administrative units had a higher average WLB score than did those employed in high-risk units (*P* < 0.001), and the managers had a lower average WLB score than did the employees without managerial positions (*P* < 0.001). The respondents who had not reported an incident within the preceding 12 months had a higher average WLB score than did those who had reported at least one incident (*P* < 0.001), and those who had no contact with patients had a higher mean WLB score than did those who have contact with patients (*P* < 0.001). Age, gender, and educational level did not affect WLB score.

### Correlations Between SAQ, EE, and WLB

As shown in Table 5, the correlation co-efficients for the SAQ subdimensions (except stress recognition) ranged from 0.65 to 0.85. The stress recognition subdimension did not exhibit a linear relationship with any of the other SAQ subdimensions. The total SAQ score was negatively correlated with the EE score and positively correlated with the WLB score. Except for the stress recognition subdimension, all the SAQ subdimensions exhibited significant negative and positive linear relationships with EE and WLB, respectively, indicating that staff members with a lower degrees of EE or greater WLB have more positive attitudes toward patient safety. The stress recognition subdimension was not significantly correlated with WLB (*r* = −0.082, *P* = 0.131) and was negatively correlated with EE (*r* = −0.230, *P* < 0.001). When an individual has low EE, their awareness of their work performance under stress will also be low. EE and WLB were not highly correlated (*r* = −0.525); therefore, when the two were simultaneously input into the regression model as independent variables, the problem of multicollinearity did not arise.

**Table 5:** Correlations matrix among dimensions of SAQ, EE, and WLB.

**Measurement**	**1**	**2**	**3**	**4**	**5**	**6**	**7**	**8**	**9**
1.Teamwork climate	1								
2.Safety climate	0.845**	1							
3.Job satisfaction	0.755**	0.809**	1						
4.Stress recognition	0.019	0.039	–0.016	1					
5.Perception of management	0.732**	0.796**	0.756**	0.021	1				
6.Working condition	0.645**	0.738**	0.706**	–0.072	0.782**	1			
7.Total SAQ score	0.861**	0.909**	0.807**	0.171**	0.826**	0.780**	1		
8.Emotional exhaustion	–0.525**	–0.548**	–0.602**	–0.230**	–0.543**	–0.569**	–0.519**	1	
9.Work-life balance	0.267**	0.317**	0.313**	–0.082	0.307**	0.418**	0.289**	–0.525**	1

^**^ P < 0.01.

### Effect of EE and WLB on SAQ

The hierarchical regression analysis results identify the factors affecting the respondents' attitudes toward patient safety culture in 2021 (see Table 6). EE and WLB were used as predictors, and demographic and professional variables (age, gender, educational level, tenure, job role, division, managerial status, number of incident reports, and whether they have contact with patients at work) served as control variables. SAQ subdimension scores were the dependent variables. WLB affects the safety climate (38), and individuals with greater WLB are less likely to experience personal burnout (39), so for the time being, low WLB occurs before burnout. Therefore, demographic and professional variables were input into the model in the first step, and WLB and EE were input into the model in the second and third steps, respectively.

**Table 6:** Hierarchical models of SAQ.

**Variables**	**Teamwork**			**Safety**			**Job**			**Stress**		**Perception of**			**Working**		
**beta**	**climate**			**climate**			**satisfaction**			**recognition**		**management**			**condition**		
**Model**	**M1**	**M2**	**M3**	**M4**	**M5**	**M6**	**M7**	**M8**	**M9**	**M10**	**M11**	**M12**	**M13**	**M14**	**M15**	**M16**	**M17**
**Control variable**																	
**Age**																	
20–40 years ^ref^	–	–	–	–	–	–	–	–	–	–	–	–	–	–	–	–	–
40–60 years	0.08	0.10	−0.03	0.12	0.14*	0.01	0.15*	0.17**	0.03	−0.11	−0.06	0.12	0.14*	0.02	0.14*	0.17	0.06
>60 years	0.24**	0.25**	0.10	0.27***	0.27***	0.12	0.38***	0.38***	0.21**	−0.14	−0.06	0.33***	0.33***	0.18**	0.30***	0.30	0.16*
**Gender** (male)	0.07	0.06	0.05	0.01	−0.01	−0.01	0.03	0.01	0.00	−0.01	0.00	0.05	0.03	0.02	0.05	0.03	0.02
**Education level**																	
High school or below ^ref^	–	–	–	–	–	–	–	–	–	–	–	–	–	–	–	–	–
Diploma or college	0.03	0.03	−0.00	0.02	0.02	−0.00	0.04	0.04	0.01	−0.05	−0.03	0.07	0.07	0.04	0.06	0.07	0.05
Graduate	−0.08	−0.08	−0.11	−0.04	−0.05	−0.07	0.02	0.02	−0.01	−0.01	0.00	0.02	0.01	−0.01	0.00	0.01	−0.01
**Tenure**																	
3 months-1 year	0.16**	0.14**	0.07	0.22**	0.18**	0.10	0.17**	0.14**	0.05	−0.01	0.04	0.20**	0.17**	0.09	0.19**	0.15**	0.08
1–4 years	0.07	0.05	−0.01	0.07	0.04	−0.03	−0.03	−0.06	−0.14	0.14*	0.18**	0.05	0.03	−0.04	0.02	0.01	−0.06
5–10 years	−0.01	−0.01	−0.07	0.04	0.04	−0.02	−0.01	−0.01	−0.08	0.06	0.09	0.00	0.01	−0.05	−0.06	−0.05	−0.10
>10 years ^ref^	–	–	–	–	–	–	–	–	–	–	–	–	–	–	–	–	–
**Profession**																	
Nurse ^ref^	–	–	–	–	–	–	–	–	–	–	–	–	–	–	–	–	–
Physician	0.05	0.03	−0.02	0.04	0.01	−0.04	0.06	0.03	−0.01	−0.14	−0.12	0.03	0.01	−0.03	0.03	0.00	−0.05
Technician	0.01	−0.03	−0.05	0.01	−0.03	−0.05	−0.04	−0.08	−0.10	−0.08	−0.06	−0.01	−0.05	−0.07	0.02	−0.03	−0.05
Administrative	0.03	0.03	−0.01	0.04	0.04	0.01	0.05	0.05	0.01	−0.19*	−0.17	0.02	0.02	−0.01	0.02	0.02	−0.02
**Division**																	
High risk units ^ref^	–	–	–	–	–	–	–	–	–	–	–	–	–	–	–	–	–
Opd/inspection units	0.29***	0.24**	0.17*	0.28***	0.21**	0.16*	0.27***	0.20**	0.13*	0.09	0.15	0.31***	0.25**	0.19**	0.28***	0.20**	0.14*
Adm. unit or other	0.04	−0.04	−0.04	0.03	−0.06	−0.07	0.05	−0.04	−0.05	0.15	−0.17*	0.13	0.04	0.04	0.17*	0.04	0.01
**Manager** (yes)	0.08	0.12*	0.11*	0.07	0.13*	0.11*	0.03	0.09	0.07	0.01	−0.00	0.00	0.04	0.03	−0.06	0.01	−0.11
**Incidient report** (yes)	0.01	0.05	0.05	0.06	0.11	0.11*	0.02	0.06	0.05	0.02	0.00	0.10	0.14*	0.14**	0.10	0.12*	0.14**
**Patient contact** (yes)	0.04	0.07	0.06	0.02	0.06	0.03	−0.02	0.01	−0.02	0.00	0.00	0.01	0.04	0.02	−0.03	−0.04	−0.12
**Predictive variable**																	
WLB	–	0.26***	0.01	–	0.33***	0.10	–	0.31***	0.05	–	–	–	0.29***	0.06	–	0.39***	0.18**
EE	–	–	−0.51***	–	–	−0.49***	–	–	−0.55***	–	0.24***	–	–	−0.48***	–	–	−0.45***
*R*-square	0.13	0.18	0.34	0.13	0.22	0.37	0.19	0.26	0.45	0.10	0.14	0.15	0.22	0.36	0.16	0.28	0.40
Adjusted *R*-square	0.09	0.14	0.30	0.09	0.17	0.33	0.15	0.22	0.42	0.06	0.10	0.11	0.17	0.32	0.12	0.24	0.36
F	2.86***	3.95***	8.67***	2.94***	5.08***	9.99***	4.66***	6.68***	14.34***	2.23**	3.14***	3.57***	5.13***	9.81***	3.70***	6.96***	11.28***
ΔR-square		0.05	0.16		0.09	0.15		0.07	0.19		0.04		0.06	0.14		0.12	0.12
ΔF		18.84***	72.77***		34.36***	73.41***		31.84***	106.90***		16.00***		25.76***	70.33***		49.73***	61.45**

*EE, emotional exhaustion; Ref, reference category; WLB, work-life balance*.

* P < 0.05, ** P < 0.01, *** P < 0.001.

The results of the hierarchical regression model (M1), in which teamwork climate was used as the dependent variable, indicate that demographic and professional variables input in the first step could jointly predict 9% of the variation in teamwork climate, and In the first model (M1), the regression model was significant. When WLB was input in the second step, it accounted for 5% of the variation in teamwork climate [Δ*R*^2^ = 0.05; F (1, 301) = 18.84, *P* < 0.001], and the result of the model (M2) was again significant. When EE was input in the third step, both WLB and EE served as predictors of teamwork climate. As a result, the explanatory power of the full model (M3) increased significantly [Δ*R*^2^ = 0.16; F (1, 300) = 72.77, *P* < 0.001], and only EE was identified as a significant predictor of teamwork climate (β= 0.51, *P* < 0.001), whereas WLB was not a predictor of teamwork climate in M3 (β= 0.01, *P* = 0.86). Other full model such as M6, M9, and M14 full models were also only EE was identified as a significant predictor, and WLB was identified as a non-significant predictor of safety climate (β= 0.10, *P* = 0.08), job satisfaction (β= 0.05, *P* = 0.38), and perception of management (β= 0.06, *P* = 0.29). M17 is the only exception, when EE was introduced in the third step, both EE and WLB exerted significant effects on working conditions (β= 0.45, *P* < 0.001 and β= 0.18, *P* = 0.001; respectively), but the effect of WLB in M17 was smaller than that in M16 (β= 0.39, *P* < 0.001).

In the full model, managerial status was a significant predictor of teamwork climate and safety climate. The managers scored higher in these two subdimensions than did the respondents without managerial positions. The respondents who had reported incidents in the preceding 12 months had higher average scores in the safety climate, perception of management, and working conditions subdimensions than did those who did not reported any incident. In addition, to account for the effects of the COVID-19 pandemic, we stratified analysis by division. According to M3, M6, M9, M14, and M17, the respondents who worked in outpatient clinics and inspection units, which tend to have high numbers of patients and short average lengths of stay, scored higher in each SAQ subdimension than did the respondents who were employed in high-risk units such as the ED, inpatient wards, and ORs (Show on M3,6,9,14,17). The respondents who were 60 years old or older had higher job satisfaction (M9), perception of management, and working conditions (M17) scores than did those who were 20–40 years old. Because no significant linear relationship was observed between stress recognition and WLB (Table 5), only EE was included in the regression model for stress recognition (M11). M11 indicated that EE was a significant predictor of stress recognition [adjusted *R*^2^ = 0.10, Δ*R*^2^ = 0.04; F (1, 318) = 16.00, *P* < 0.001]. To summarize, higher EE is associated with greater stress recognition.

## Discussion

By 2021, a year after the onset of the COVID-19 pandemic, the respondents' EE and WLB scores had improved significantly. WLB positively affected scores in the SAQ subdimensions of teamwork climate, safety climate, job satisfaction, perception of management, and working conditions, and EE exerted the strongest effect on the SAQ all subdimension during the COVID-19 Pandemic.

### Changes in Patient Safety Culture During the Epidemic

No significant difference was identified between the respondents' 2020 and 2021 average patient safety attitude scores. Effective communication was determined to affect patient safety culture in previous studies (32, 35, 40). From the beginning of the COVID-19 pandemic in 2020 to the present, the implementation of comprehensive infection control interventions mandating the use of personal protective equipment that covers most of the face has increased the complexity of interpersonal communication (41). In addition, Strict regulations related to infection control undermine mutual support among hospital staff by preventing staff members from helping each other with certain tasks (42).

However, the COVID-19 pandemic has cultivated positive opportunities for interprofessional interactions and teamwork among hospital staff (43), including interdepartmental support and collaboration on tasks in response to policy or outbreak developments, such as the construction of quarantine sites at the entrance of the hospital in 2020 and the implementation of vaccination programs in 2021, both of which were resource-intensive projects (especially for small hospitals).

None of the average SAQ subdimension scores differed significantly between 2020 and 2021. However, according to the cut-off point of 75 points stipulated by the JCT, the attitudes of the employees toward safety climate, job satisfaction, and perception of management changed from negative to positive from 2020 to 2021. The COVID-19 pandemic has forced hospital workers to acknowledge their workplace as a high-risk environment and to abide by various pandemic prevention measures, thereby improving safety awareness and, in turn, patient safety culture.

Sreeramoju et al. (44) adopted a positive deviance approach in their study exploring the social aspects of infection prevention practices, which demonstrated the importance of identifying local role models for accelerating change and developing actionable solutions, which, in turn, strongly affect patient safety climate. Such approaches consistently emphasize strengthening the awareness of patient safety within the hospital, learning through interaction with exemplary role models, and promoting stress management among peers, thereby having positivity about the work experience; these positive attitudes may be reflected in employees' job satisfaction subdimension scores becoming positive These positive attitudes about work experience result from the accumulation of knowledge of and practical experience in dealing with COVID-19, allowing staff to feel autonomous in organizing patient care in the best possible way (42). In this study, because WLB affects safety climate, the positive shift in attitudes regarding safety climate may also be the attributable to an improvement in WLB in 2021.

The respondents' WLB and EE scores improved from 2020 to 2021. EE is the core element of burnout, and it reflects individuals' stress levels (45).At the onset of the COVID-19 pandemic in 2020, medical professionals were under increased pressure from multiple sources, including increased workload, fear of bringing the virus home, possible infection, inability to deal with patients refusing to cooperate with medical procedures, and fear of dealing with patients' emotional issues (such as anxiety and panic), fear of protective equipment shortages putting them at risk when treating critical patients, the need to adapt to frequently changing policies, and obligations to family members and others outside the hospital (22, 41).

Among the problems mentioned above, shortages of personal protective equipment are of particular concern to hospital staff (46), and the difficulty in purchasing protective equipment and the rising prices of such equipment were major challenges faced by hospital managers in the early stages of the pandemic (34). However, with the unified procurement and regulation of masks implemented by the Taiwanese government on January 30, 2020, stress from the Acquisition of materials was slightly alleviated despite the continuing supply shortage. More time could be spent on epidemic prevention. The average workload had decreased due to the cancellation of non-essential surgeries, which resulted from patients' fear of being infected at a hospital (47).

May 2021 was the peak of the COVID-19 pandemic in Taiwan. The Taiwan Centers for Disease Control regulated medical institutions to reduce the workload and instructed such institutions to suspend medical treatments that could be post-poned. In addition, because the hospital in our study was a district hospital, but not a hospital dedicated to COVID-19 patients, the stress of the staff was low, possibly resulting in lower EE scores in August 2021. Furthermore, with continuous education, training, and public awareness efforts regarding the transmission routes and pathogenic mechanisms of COVID-19 and with the provision of infection prevention-related information, medical staff became more familiar with emerging infectious diseases and related treatment procedures. The staff tended to have a higher degree of positive WLB because of the lower workload and fewer shifts in May 2021. Some of the hospital staff had begun dividing their work between home and hospital, which enabled them to manage their work and their family responsibilities, including children who may have been studying online at home due to the suspension of classes.

### Predictors of Patient Safety Culture

Although the predictive value of demographic and professional variables for SAQ subdimension scores was not the focus of this study, the results showed that the respondents employed in outpatient and examination units, which tend to have the highest patient number, had the highest average scores for every SAQ subdimension, except stress recognition. The employees' sensitivity to patient safety had increased because the staff were under frequent exposure to asymptomatic patients and were therefore required to observe strict infection control measures.

Some of the demographic and professional variables exhibited significant predictive power for each SAQ subdimension in the full regression model, which differs from the results reported by Chen et al. (22). This is mainly attributable to the distinct sorting methods used for demographic variables. For example, this study had four categories for the age variable, with three dummy variables, whereas the study by Chen et al. had only two age groups, and the other categorical variables were also dichotomized.

Incident reporting is a critical component of patient safety culture (32). In the present study, the respondents who had reported incidents within the preceding 12 months had higher average scores in the perception of management and working conditions sub-dimensions than did those who had not reported any incident.

Stress recognition was the only subdimension of patient safety culture that did not exhibit a linear relationship with WLB. EE was determined to negatively affect stress recognition, which is consistent with the results of a study on community nurses, which considered high stress recognition scores to be a reflection of longer on-call hours (48), which may be associated with greater EE.

However, scholars using confirmatory factor analysis have reported that stress recognition was a strong one-factor model, and that it is only weakly correlated (*r* = −0.15 to 0.03) with the other five sub-dimensions of the SAQ, indicating that the stress recognition subscale does not fit into the overall safety climate construct in the SAQ, which was designed to reflect safety climate (49). In this study, the correlations between stress recognition and each of the other sub-dimensions ranged from −0.02 to 0.02 (*P* > 0.05), which is similar to the results reported by Taylor and Pandian (49). Stress recognition is the only subdimension of the SAQ that accounts for personal behavior and is affected by many confounding factors (35); therefore, it will not be discussed further the statistical test results related to them in this paper.

Finally, regarding the theoretical basis of the present study, the hierarchical regression test revealed that after demographic and professional variables were controlled for, WLB could predict all SAQ sub-dimensions (except for stress recognition). However, when EE was incorporated into the model, WLB lost its predictive power, which may be because some of the information accounted for by the WLB scale overlapped with that accounted for by the EE scale.

Although a large-scale study indicated that the effect of WLB on the safety climate is achieved entirely through the full mediation of EE and teamwork climate, district hospital staff accounted for only 3.2% of the sample of the study, and the study focused on ICUs, EDs, and ORs (38).

### Limitations

Although this study adopted a robust research design, it still has some limitations. First, the study conducted an in-depth analysis of the changes in the patient safety culture as the COVID-19 pandemic progressed. However, it only used variables employed by the JCT and could not, therefore, evaluate the effects of patient safety culture, such as workforce load or employee engagement in patient safety culture. Prospective studies should be conducted in the future. Second, the generalizability of the study results is limited by the small sample of physicians serving as frontline caregivers during the pandemic and the collection of the study data from a single regional hospital in Taiwan.

## Conclusions

This study investigated the changes in patient safety culture in a regional hospital during the COVID-19 pandemic. Health-care professionals employed at the hospital have faced numerous challenges related to the COVID-19 pandemic, such as those related to redeployment of district hospital operators. From 2020 to 2021, the employees' attitudes in three SAQ sub-dimensions—safety climate, job satisfaction, and perception of management—changed from negative to positive. In addition, to preserve medical capacity, the government reduced the workload of health-care professionals, reducing consultations with doctors for psychological conditions. With the decreased labor demand and diversion of workload, EE and WLB significantly improved, and the study results indicate that both EE and WLB are key factors affecting patient safety culture. The results of this study can serve as a reference for hospital managers to develop plans for responding to Crises, which integrate appropriate education, information transparency, and training to motivate staff to participate in learning from incident event, to actively promote patient safety, to exhibit concern for internal issues, and to engage in specific problem solving. A positive patient safety culture can be cultivated with reasonable working hours and effective communication.

## Data Availability Statement

The raw data supporting the conclusions of this article will be made available by the authors, without undue reservation.

## Ethics Statement

The studies involving human participants were reviewed and approved by CMUH111-REC-001. Written informed consent for participation was not required for this study in accordance with the national legislation and the institutional requirements.

## Author Contributions

SJW, YCC, and WYH: study conception and design. SJW and YHS: data collection. All authors: data analysis and interpretation, drafting of the article, and critical revision of the article. All authors contributed to the article and approved the submitted version.

## Conflict of Interest

The authors declare that the research was conducted in the absence of any commercial or financial relationships that could be construed as a potential conflict of interest.

## Publisher's Note

All claims expressed in this article are solely those of the authors and do not necessarily represent those of their affiliated organizations, or those of the publisher, the editors and the reviewers. Any product that may be evaluated in this article, or claim that may be made by its manufacturer, is not guaranteed or endorsed by the publisher.

## References

[1] Alagha MA Jaulin F Yeung W Celi LA Cosgriff CV Myers LC. Patient harm during Covid-19 pandemic: using a human factors lens to promote patient and workforce safety. J Patient Saf. (2021) 17:87–9. 10.1097/PTS.0000000000000798 33273400 PMC8034827

[2] Mashi MS Subramaniam C Johari J Suleiman Abubakar S. Understanding safety management practices and safety performance amid coronavirus (Covid-19) pandemic among nurses in public hospitals. Int J Public Admin. (2021):1–12. 10.1080/01900692.2021.2012803. [Epub ahead of print].

[3] Wang Y He Y Sheng Z Yao X. When does safety climate help? A multilevel study of Covid-19 risky decision making and safety performance in the context of business reopening. J Bus Psychol. (2022):1–15. 10.1007/s10869-022-09805-3. [Epub ahead of print]. 35310340 PMC8922079

[4] Dal Corso L. Mediation effects of safety climate and safety motivation on the relation between organizational climate and safety performance in the workplace. TPM Test Psychom Methodol Appl Psychol. (2008) 15:77–90. 10.4473/TPM.15.2.2

[5] Moore D Gamage B Bryce E Copes R Yassi A Group BIRPS. Protecting health care workers from sars and other respiratory pathogens: organizational and individual factors that affect adherence to infection control guidelines. Am J Infect Control. (2005) 33:88–96. 10.1016/j.ajic.2004.11.003 15761408 PMC7115321

[6] De Vries EN Ramrattan MA Smorenburg SM Gouma DJ Boermeester MA. The incidence and nature of in-hospital adverse events: a systematic review. BMJ Qual Saf. (2008) 17:216–23. 10.1136/qshc.2007.023622 18519629 PMC2569153

[7] Sousa P Uva AS Serranheira F Nunes C Leite ES. Estimating the incidence of adverse events in Portuguese hospitals: a contribution to improving quality and patient safety. BMC Health Serv Res. (2014) 14:1–6. 10.1186/1472-6963-14-311 25034870 PMC4114085

[8] Ferorelli D Benevento M Vimercati L Spagnolo L De Maria L Caputi A. Improving healthcare workers' adherence to surgical safety checklist: the impact of a short training. Front Public Health. (2021) 9:732707. 10.3389/fpubh.2021.732707 35211450 PMC8860967

[9] Weaver SJ Lubomksi LH Wilson RF Pfoh ER Martinez KA Dy SM. Promoting a culture of safety as a patient safety strategy: a systematic review. Ann Intern Med. (2013) 158(5_Part_2):369–74. 10.7326/0003-4819-158-5-201303051-00002 23460092 PMC4710092

[10] AbuAlRub RF Abu Alhijaa EH. The impact of educational interventions on enhancing perceptions of patient safety culture among Jordanian senior nurses. Nurs Forum. (2014) 49:139–50. 10.1111/nuf.12067 24392690

[11] Pronovost PJ Berenholtz SM Goeschel CA Needham DM Sexton JB Thompson DA. Creating high reliability in health care organizations. Health Serv Res. (2006) 41(4p2):1599–617. 10.1111/j.1475-6773.2006.00567.x 16898981 PMC1955341

[12] Rodziewicz TL Houseman B Hipskind JE. Medical Error Reduction Prevention. In: StatPearls [Internet]. Treasure Island (FL): StatPearls Publishing (2022). Available online at: https://www.ncbi.nlm.nih.gov/books/NBK499956/29763131

[13] Cooper MD. Toward a model of safety culture. Safety Sci. (2000) 36:111–36. 10.1016/S0925-7535(00)00035-7

[14] Institute of Medicine. To Err Is Human: Building a Safer Health System. Washington, DC: The National Academies Press (US) (2000). 10.17226/9728 25077248

[15] Okuyama JHH Galvao TF Silva MT. Healthcare professional's perception of patient safety measured by the hospital survey on patient safety culture: a systematic review and meta-analysis. ScientificWorldJournal. (2018) 2018:9156301. 10.1155/2018/9156301 30104917 PMC6076892

[16] Di Muzio M Dionisi S Di Simone E Cianfrocca C Di Muzio F Fabbian F. Can nurses' shift work jeopardize the patient safety? A systematic review. Eur Rev Med Pharmacol Sci. (2019) 23:4507–19. 10.26355/eurrev_201905_17963 31173328

[17] DiCuccio MH. The relationship between patient safety culture and patient outcomes: a systematic review. J Patient Saf. (2015) 11:135–42. 10.1097/PTS.0000000000000058 24583952

[18] Dodek PM Wong H Heyland DK Cook DJ Rocker GM Kutsogiannis DJ. The relationship between organizational culture and family satisfaction in critical care. Crit Care Med. (2012) 40:1506–12. 10.1097/CCM.0b013e318241e368 22511132

[19] Health and Safety Commission. ACSNI Study Group on Human Factors. 3rd Report. Organizing for Safety. London: HSC (1993)

[20] Li N Marshall D Sykes M McCulloch P Shalhoub J Maruthappu M. Systematic review of methods for quantifying teamwork in the operating theater. BJS Open. (2018) 2:42–51. 10.1002/bjs5.40 29951628 PMC5952378

[21] Sexton JB Helmreich RL Neilands TB Rowan K Vella K Boyden J. The safety attitudes questionnaire: psychometric properties, benchmarking data, and emerging research. BMC Health Serv Res. (2006) 6:1–10. 10.1186/1472-6963-6-44 16584553 PMC1481614

[22] Chen HY Lu L Ko YM Chueh JW Hsiao SY Wang PC. Post-pandemic patient safety culture: a case from a large metropolitan hospital group in Taiwan. Int J Env Res Pub Health. (2021) 18:4537. 10.3390/ijerph18094537 33923352 PMC8123150

[23] Yu B Wen C-F Lo H-L Liao H-H Wang P-C. Improvements in patient safety culture: a national Taiwanese survey, 2009–16. Int J Qual Health Care. (2020) 32:A9–17. 10.1093/intqhc/mzz099 31917449

[24] Davenport DL Henderson WG Mosca CL Khuri SF Mentzer Jr RM. Risk-adjusted morbidity in teaching hospitals correlates with reported levels of communication and collaboration on surgical teams but not with scale measures of teamwork climate, safety climate, or working conditions. J Am Coll Surgeons. (2007) 205:778–84. 10.1016/j.jamcollsurg.2007.07.039 18035261

[25] Gómez-Ochoa SA Franco OH Rojas LZ Raguindin PF Roa-Díaz ZM Wyssmann BM. Covid-19 in health-care workers: a living systematic review and meta-analysis of prevalence, risk factors, clinical characteristics, and outcomes. Am J Epidemiol. (2021) 190:161–75. 10.1093/aje/kwaa191 32870978 PMC7499478

[26] Raudenská J Steinerová V Javurková A Urits I Kaye AD Viswanath O. Occupational burnout syndrome and post-traumatic stress among healthcare professionals during the novel coronavirus disease 2019 (Covid-19) pandemic. Best Pract Res Clin Anaesthesiol. (2020) 34:553–60. 10.1016/j.bpa.2020.07.008 33004166 PMC7367798

[27] Ferorelli D Mandarelli G Solarino B. Ethical challenges in health care policy during Covid-19 pandemic in Italy. Medicina. (2020) 56:691. 10.3390/medicina56120691 33322462 PMC7764230

[28] Al Ma'mari Q Sharour LA Al Omari O. Fatigue, burnout, work environment, workload and perceived patient safety culture among critical care nurses. Br J Nurs. (2020) 29:28–34. 10.12968/bjon.2020.29.1.28 31917951

[29] Voydanoff P. Linkages between the work-family interface and work, family, and individual outcomes: an integrative model. J Fam Issues. (2002) 23:138–64. 10.1177/0192513X02023001007

[30] Guest DE. Perspectives on the study of work-life balance. Soc Sci Inform. (2002) 41:255–79. 10.1177/0539018402041002005

[31] Sammer CE Lykens K Singh KP Mains DA Lackan NA. What is patient safety culture? A review of the literature. J Nurs Scholarship. (2010) 42:156–65. 10.1111/j.1547-5069.2009.01330.x 20618600

[32] Kumbi M Hussen A Abate Lette SN Morka G. Patient safety culture and associated factors among health care providers in bale zone hospitals, Southeast Ethiopia: an institutional based cross-sectional study. Drug Healthc Patient Saf. (2020) 12:1. 10.2147/DHPS.S198146 32021477 PMC6971344

[33] Denning M Goh ET Scott A Martin G Markar S Flott K. What has been the impact of Covid-19 on safety culture? A case study from a large metropolitan healthcare trust. Int J Env Res Pub Health. (2020) 17:7034. 10.3390/ijerph17197034 32993013 PMC7579589

[34] Grimm CA. Hospital Experiences Responding to the Covid-19 Pandemic: Results of a National Pulse Survey March 23–27, 2020. Washington, DC: US Department of Health and Human Services Office of Inspector General (2020). p. 41. 10.1002/ev.1561

[35] Nguyen G Gambashidze N Ilyas SA Pascu D. Validation of the safety attitudes questionnaire (Short Form 2006) in Italian in hospitals in the northeast of Italy. BMC Health Serv Res. (2015) 15:1–8. 10.1186/s12913-015-0951-8 26204957 PMC4512154

[36] Bulajić M Lazibat T Plavec D. Validation of the safety attitudes questionnaire (Short Form 2006) in management staff of Croatian hospitals. Bus Excell. (2018) 12:55–71. 10.22598/pi-be/2018.12.1.55

[37] Maslach C Jackson SE. The measurement of experienced burnout. J Organ Behav. (1981) 2:99–113. 10.1002/job.4030020205

[38] Tran Y Liao HH Yeh EH Ellis LA Clay-Williams R Braithwaite J. Examining the pathways by which work-life balance influences safety culture among healthcare workers in Taiwan: path analysis of data from a cross-sectional survey on patient safety culture among hospital staff. BMJ Open. (2021) 11:e054143. 10.1136/bmjopen-2021-054143 34728459 PMC8565544

[39] Schwartz SP Adair KC Bae J Rehder KJ Shanafelt TD Profit J. Work-life balance behaviors cluster in work settings and relate to burnout and safety culture: a cross-sectional survey analysis. BMJ Qual Saf. (2019) 28:142–50. 10.1136/bmjqs-2018-007933 30309912 PMC6365921

[40] El-Jardali F Dimassi H Jamal D Jaafar M Hemadeh N. Predictors and outcomes of patient safety culture in hospitals. BMC Health Serv Res. (2011) 11:1–12. 10.1186/1472-6963-11-45 21349179 PMC3053221

[41] Walton M Murray E Christian MD. Mental health care for medical staff and affiliated healthcare workers during the Covid-19 pandemic. Eur Heart J Acute Cardiovasc Care. (2020) 9:241–7. 10.1177/2048872620922795 32342698 PMC7189614

[42] van der Goot WE Duvivier RJ Van Yperen NW de Carvalho-Filho MA Noot KE Ikink R. Psychological distress among frontline workers during the Covid-19 pandemic: a mixed-methods study. PLoS ONE. (2021) 16:e0255510. 10.1371/journal.pone.0255510 34351970 PMC8341539

[43] Goldman J Xyrichis A. Interprofessional working during the Covid-19 pandemic: sociological insights. J Interprof Care. (2020) 34:580–2. 10.1080/13561820.2020.1806220 32838586

[44] Sreeramoju P Dura L Fernandez ME Minhajuddin A Simacek K Fomby TB. Using a positive deviance approach to influence the culture of patient safety related to infection prevention. Open Forum Infect Dis. (2018) 5:ofy231. 10.1093/ofid/ofy231 30288392 PMC6166267

[45] Maslach C Schaufeli WB Leiter MP. Job burnout. Annu Rev Psychol. (2001) 52:397–422. 10.1146/annurev.psych.52.1.397 11148311

[46] Dong Z-Q Ma J Hao Y-N Shen X-L Liu F Gao Y. The social psychological impact of the Covid-19 pandemic on medical staff in China: a cross-sectional study. Eur Psychiat. (2020) 63:1–8. 10.1192/j.eurpsy.2020.59 32476633 PMC7343668

[47] Søreide K Hallet J Matthews JB Schnitzbauer AA Line PD Lai P. Immediate and long-term impact of the Covid-19 pandemic on delivery of surgical services. Br J Surg. (2020) 107:1250–61. 10.1002/bjs.11670 32350857 PMC7267363

[48] Yoshida Y Sandall J. Occupational burnout and work factors in community and hospital midwives: a survey analysis. Midwifery. (2013) 29:921–6. 10.1016/j.midw.2012.11.002 23415363

[49] Taylor JA Pandian R. A dissonant scale: stress recognition in the saq. BMC Res Notes. (2013) 6:1–6. 10.1186/1756-0500-6-302 23902850 PMC3733616

